# Supplementary material for: The core and accessory Hfq interactomes across Pseudomonas aeruginosa lineages
Source: Nat Commun. 2022 Mar 10;13:1258. doi: 10.1038/s41467-022-28849-w (PMC8913705; doi:10.1038/s41467-022-28849-w)
Supplement: Supplementary file 1 — Supplementary Information [file 41467_2022_28849_MOESM1_ESM.pdf]

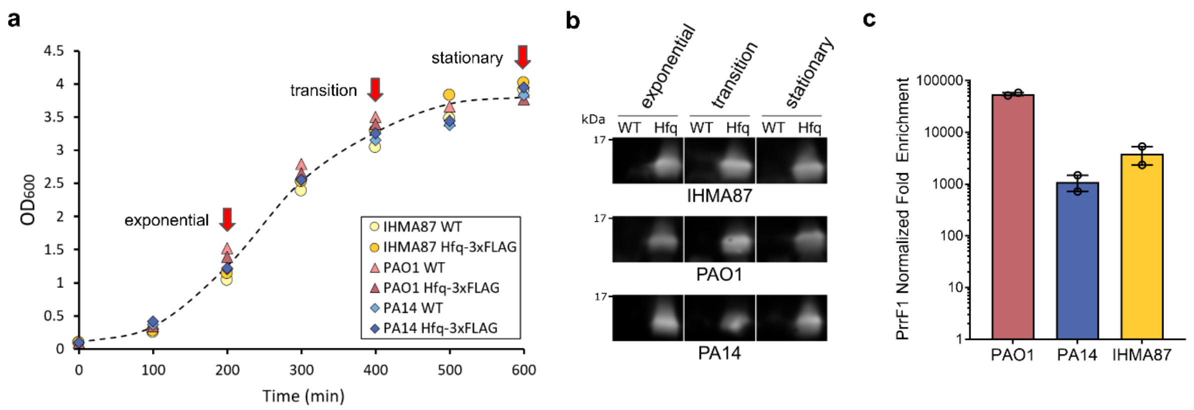

Supplementary Fig. 1. RIP-seq experimental procedure. (**a**) Growth curves of the strains used in this study in LB medium at 37°C. Sampling time points are denoted by red arrows. (**b**) Western Blot analysis of Hfq across strains and time points. Whole bacteria were sampled and analyzed by SDS-PAGE followed by anti-FLAG antibody immunodetection. Results are from one representative experiment out of three. (**c**) RT-qPCR analysis of PrrF1 co-Immunoprecipitation with Hfq in three strains. All strains were grown in LB medium for 8 hours in biological duplicates. Enrichment between Hfq-3xFLAG and WT strains were normalized to the abundance of 6S RNA. Error bars indicate the s.e.m..

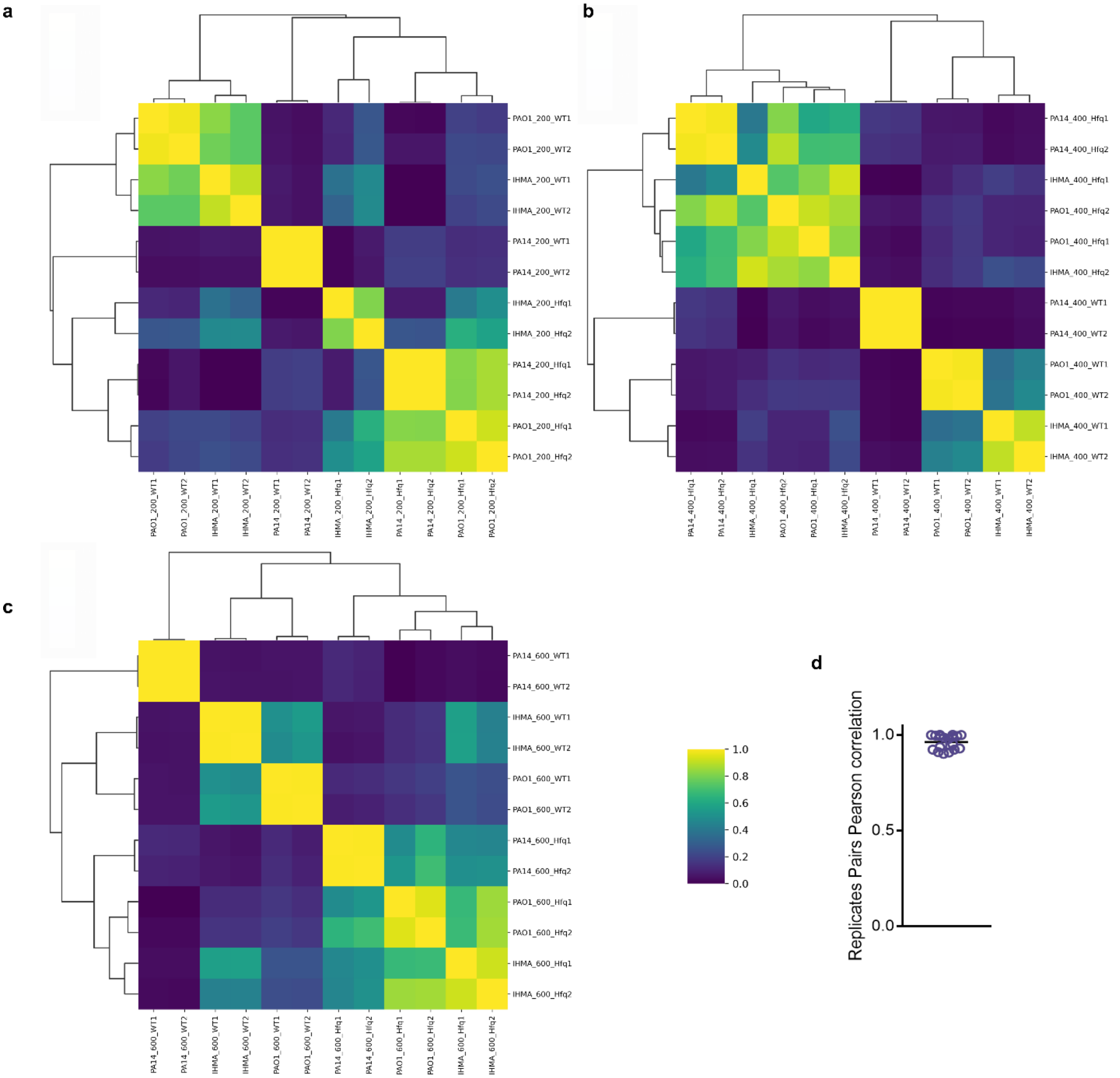

Supplementary Fig. 2. Replicates correlation analysis. (**a-c**) Hierarchical clustering and heatmap of Pearson correlation coefficient between all biological replicates based on features counts from genes conserved in all three strains for exponential (**a**), transition (**b**) and stationary (**c**) phases. (**d**) Pearson correlation coefficient between paired biological replicates.

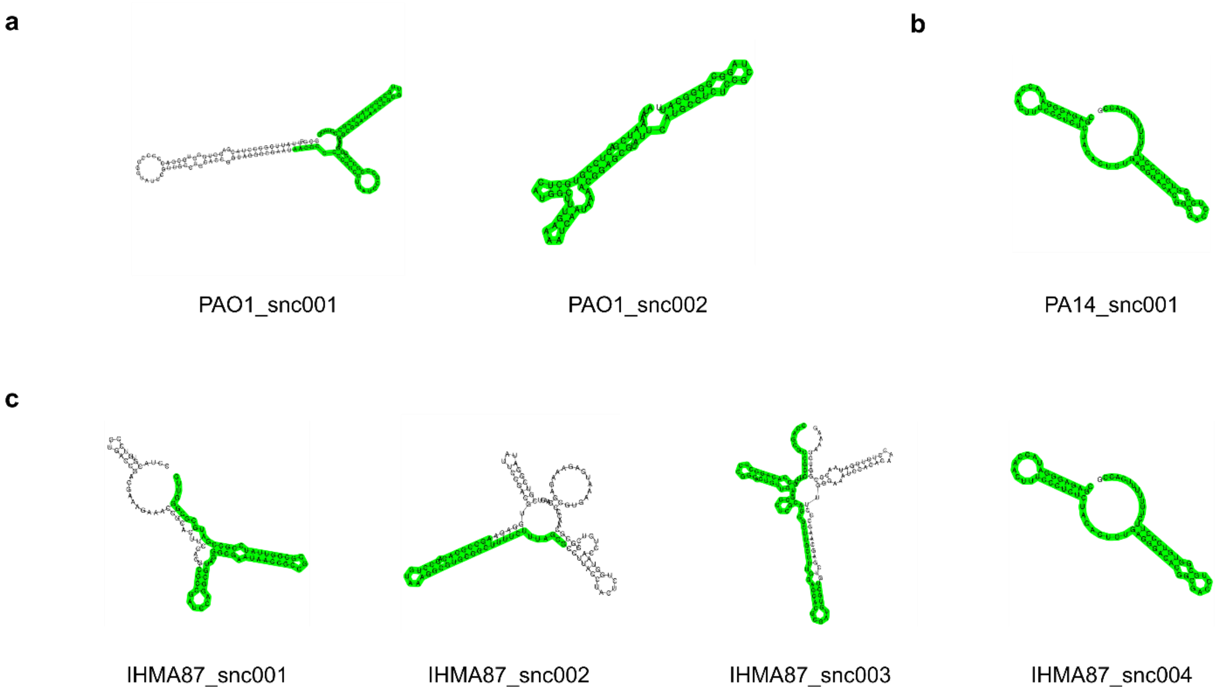

Supplementary Fig. 3. The analysis of intergenic peaks reveals potential new ncRNA motifs. Strictly intergenic peaks were used to detect known ncRNA motifs using StructRNAfinder (https://structrnafinder.integrativebioinformatics.me) in PAO1 (**a**), PA14 (**b**) and IHMA87 (**c**). ncRNA annotations correspond to the IDs found in Supplementary Table 5. Nucleotides highlighted in green correspond to conserved positions with StructRNAfinder database motifs.

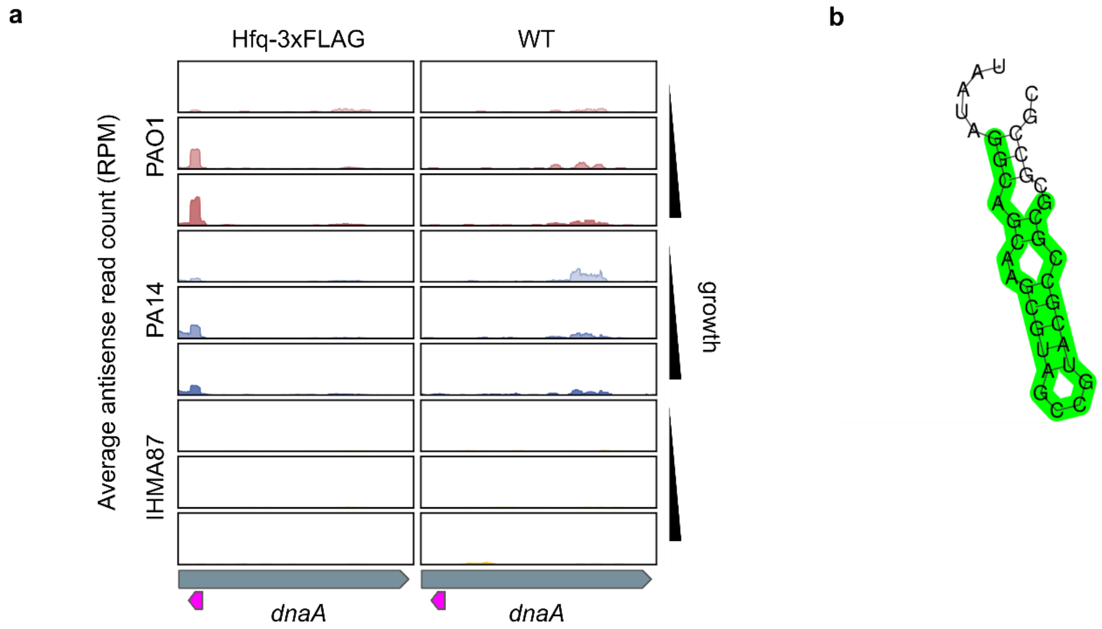

Supplementary Fig. 4. Detection of a new antisense ncRNA in the *dnaA* gene. (**a**) Antisense read coverages, averaged between biological replicates, at the *dnaA* locus for Hfq-tagged and control samples in all strains and growth phases. The new antisense potential ncRNA is denoted with a pink arrow. (**b**) ncRNA motif detected with StructRNAfinder in the *dnaA* antisense peak.

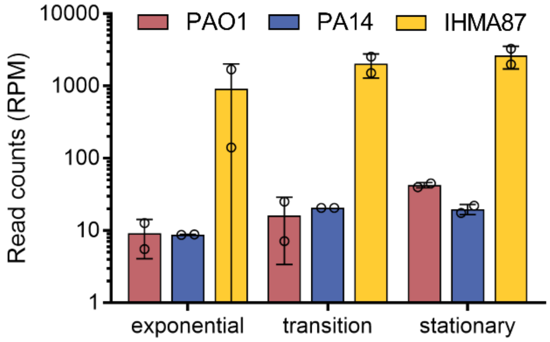

Supplementary Fig. 5. The *pvdA* RNA is much more abundant in the IHMA87 strain. Normalized read counts for *pvdA* in RIP-seq control samples shown as mean ± SD for two biological replicates. Read counts are expressed in read per million reads (RPM).

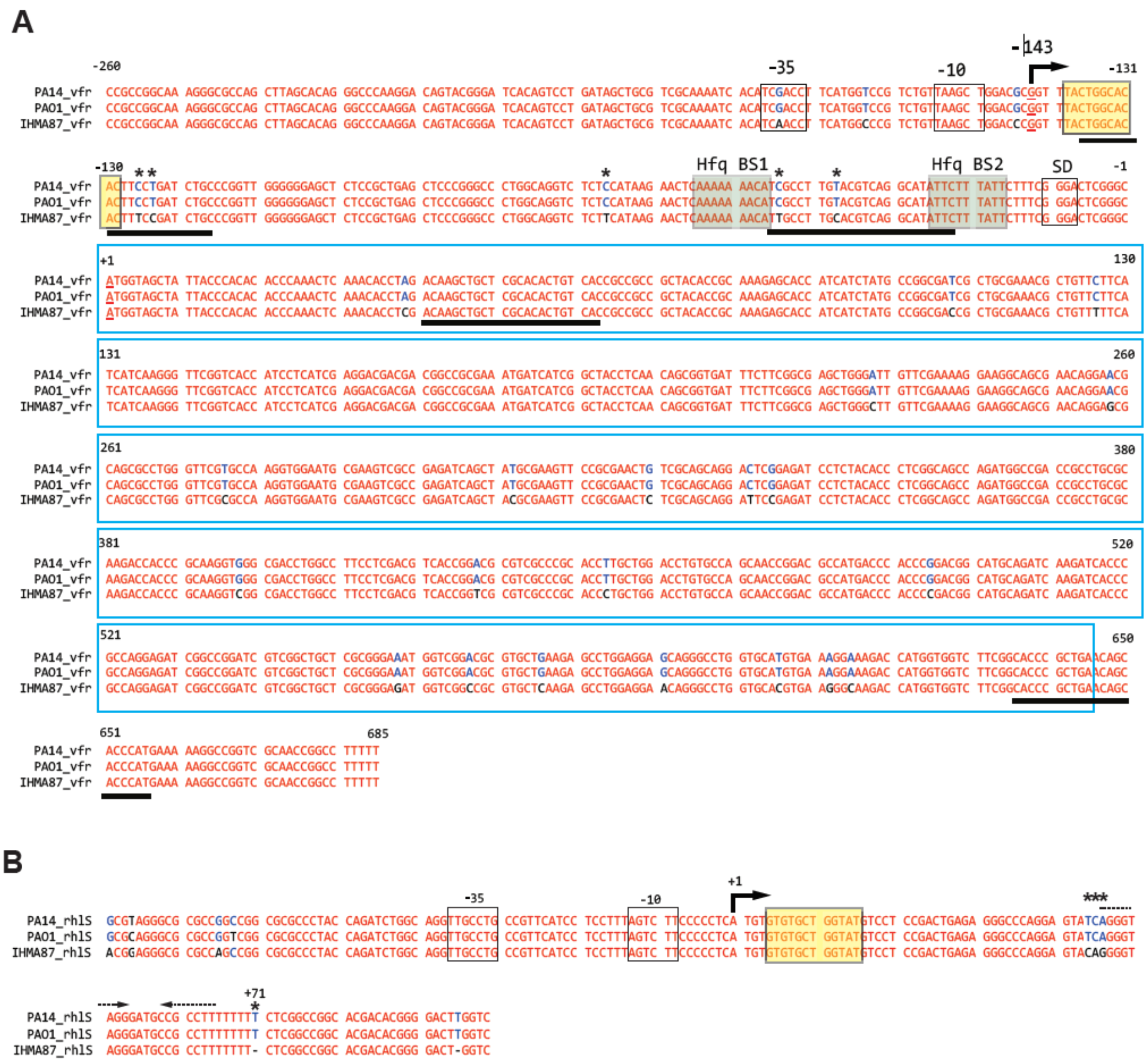

Supplementary Fig. 6. The DNA sequence alignment of *vfr* and *rhlS* genes in *P. aeruginosa* strains PA14, PAO1, and IHMA87 using Multalin (http://multalin.toulouse.inra.fr/multalin). (**a**) DNA sequence alignment of *vfr* and its regulatory region. The CDS (coding sequence) of *vfr* is shown in the boxes with blue lines. The arrow indicates the transcriptional start site relative to the nucleotide A (+1) of translation start codon. The four lines below *vfr* gene represent the locations corresponding to the binding sites by the multiple complementary oligonucleotides used in the enrichment of *vfr* chimeras. RhlS-binding site in 5’ UTR (untranslated region) of *vfr* mRNA is shown in yellow box. The 5 nucleotide differences in the *vfr* 5' UTR between IHMA87 and the two others are marked with stars (*). The two putative Hfq binding sites, Hfq BS1 and Hfq BS2, that are suggested by Irie et al. are shown in two green boxes. SD, Shine-Dalgarno sequence. (**b**) The alignment of the RhlS sequences. *vfr*-binding site of RhlS is shown in yellow box. The terminator of RhlS is shown by the dotted arrows. The stars (*) show the nucleotide differences between the RhlS sequence of IHMA87 and PA14 and PAO1.
