# Supplementary material for: The core and accessory Hfq interactomes across Pseudomonas aeruginosa lineages
Source: Nat Commun. 2022 Mar 10;13:1258. doi: 10.1038/s41467-022-28849-w (PMC8913705; doi:10.1038/s41467-022-28849-w)
Supplement: Supplementary file 2 — Reporting Summary [file 41467_2022_28849_MOESM2_ESM.pdf]

nature research

Corresponding author(s): Stephen Lory

Last updated by author(s): Feb 7, 2022

Reporting Summary

Nature Research wishes to improve the reproducibility of the work that we publish. This form provides structure for consistency and transparency in reporting. For further information on Nature Research policies, see our Editorial Policies and the Editorial Policy Checklist.

Statistics

For all statistical analyses, confirm that the following items are present in the figure legend, table legend, main text, or Methods section.

n/a	Confirmed	
☐	☒	The exact sample size (n) for each experimental group/condition, given as a discrete number and unit of measurement
☐	☒	A statement on whether measurements were taken from distinct samples or whether the same sample was measured repeatedly
☐	☒	The statistical test(s) used AND whether they are one- or two-sided<br>*Only common tests should be described solely by name; describe more complex techniques in the Methods section.*
☐	☒	A description of all covariates tested
☐	☒	A description of any assumptions or corrections, such as tests of normality and adjustment for multiple comparisons
☐	☒	A full description of the statistical parameters including central tendency (e.g. means) or other basic estimates (e.g. regression coefficient) AND variation (e.g. standard deviation) or associated estimates of uncertainty (e.g. confidence intervals)
☐	☒	For null hypothesis testing, the test statistic (e.g. F, t, r) with confidence intervals, effect sizes, degrees of freedom and P value noted<br>*Give P values as exact values whenever suitable.*
☐	☒	For Bayesian analysis, information on the choice of priors and Markov chain Monte Carlo settings
☐	☒	For hierarchical and complex designs, identification of the appropriate level for tests and full reporting of outcomes
☐	☒	Estimates of effect sizes (e.g. Cohen's d, Pearson's r), indicating how they were calculated

Our web collection on statistics for biologists contains articles on many of the points above.

Software and code

Policy information about availability of computer code

Data collection	No software was used for data collection.
Data analysis	RIP-seq data was analyzed using Trimmomatic (Galaxy version 0.38.0), Bowtie2 (Galaxy version 2.3.4.3), htseq-count (Galaxy version 0.9.1), DESeq2 (Galaxy version 2.11.40.6), different tools from BEDTools and PEAKachu version 0.1.0.2.<br>rGRIL-seq data was analyzed using CLC Genomic Workbench 7.0.<br>Homolog search were performed with BLAST reciprocal best hits (Galaxy Version 0.1.11) or megablast (blastn Galaxy version 0.3.3), multiple alignments with MAFFT (Galaxy version 7.221.3) and phylogenetic analyses with MEGA X and iTOL v5.<br>Functional enrichment analyses were performed using DAVID v6.8. Hierarchical clustering analyses were performed with BioVinci v1.1.5.<br>CRISPR spacer prediction and annotation were performed using CRISPRCasFinder and CRISPRTarget.<br>RNA structure and interaction predictions were done using StructRNAfinder and IntaRNA, respectively.<br>Data display and statistical analysis were performed with GraphPad Prism 7.04 and Python 3.7.

For manuscripts utilizing custom algorithms or software that are central to the research but not yet described in published literature, software must be made available to editors and reviewers. We strongly encourage code deposition in a community repository (e.g. GitHub). See the Nature Research guidelines for submitting code & software for further information.

Data

Policy information about availability of data

All manuscripts must include a data availability statement. This statement should provide the following information, where applicable:

- Accession codes, unique identifiers, or web links for publicly available datasets
- A list of figures that have associated raw data
- A description of any restrictions on data availability

RIP-seq and rGRIL-seq data are available under the GEO accession numbers GSE171056 [https://www.ncbi.nlm.nih.gov/geo/query/acc.cgi?acc=GSE171056] and GSE171893 [https://www.ncbi.nlm.nih.gov/geo/query/acc.cgi?acc=GSE171893], respectively. Genomes annotations are from the Pseudomonas database [https://www.pseudomonas.com/] (IHMA87 genome under the ID AZPAE15042). Source data are provided with this paper.

Field-specific reporting

Please select the one below that is the best fit for your research. If you are not sure, read the appropriate sections before making your selection.

☒ Life sciences ☐ Behavioural & social sciences ☐ Ecological, evolutionary & environmental sciences

For a reference copy of the document with all sections, see nature.com/documents/nr-reporting-summary-flat.pdf

Life sciences study design

All studies must disclose on these points even when the disclosure is negative.

Sample size	Samples sizes were not predetermined using statistical methods but were chosen based on the standards in the field.
Data exclusions	No data was excluded.
Replication	Number of replicates per experiment are described in each figure legend or in the main text.
Randomization	Randomization was not needed as the results of the different approaches used are not impacted by sequence order.
Blinding	No blinding was used as data analyses were automated and standard for all samples.

Reporting for specific materials, systems and methods

We require information from authors about some types of materials, experimental systems and methods used in many studies. Here, indicate whether each material, system or method listed is relevant to your study. If you are not sure if a list item applies to your research, read the appropriate section before selecting a response.

Materials & experimental systems

n/a	Involved in the study	
☐	☒	Antibodies
☒	☐	Eukaryotic cell lines
☒	☐	Palaeontology and archaeology
☒	☐	Animals and other organisms
☒	☐	Human research participants
☒	☐	Clinical data
☒	☐	Dual use research of concern

Methods

n/a	Involved in the study	
☒	☐	ChIP-seq
☒	☐	Flow cytometry
☒	☐	MRI-based neuroimaging

Antibodies

Antibodies used	anti-Vfr - developed and validated by Attrée lab<br>anti-FliC - developed and validated by Attrée lab<br>anti-FLAG M2 - Sigma, cat# F1804<br>anti-mouse-HRP - Sigma, cat# A9044<br>anti-rabbit-HRP - Sigma, cat# A0545
Validation	Antibodies were validated with isogenic mutants in the Attrée lab. Anti-FLAG antibodies were validated for correct immunoprecipitation of tagged proteins, along with untagged control proteins.
