# Supplementary material for: The core and accessory Hfq interactomes across Pseudomonas aeruginosa lineages
Source: Nat Commun. 2022 Mar 10;13:1258. doi: 10.1038/s41467-022-28849-w (PMC8913705; doi:10.1038/s41467-022-28849-w)
Supplement: Supplementary file 4 — Description of Additional Supplementary Files [file 41467_2022_28849_MOESM4_ESM.pdf]

Title: Supplementary Data 1.
Description: RIP-seq differential analysis results. Fold changes and q-values from the DESeq2 analysis are reported for each strain and growth condition. Genes are shown as homolog groups with the corresponding gene IDs from the three genomes. No gene IDs means the gene is not present in the corresponding genome. Gene names and COGs are from PAO1 annotation on the Pseudomonas database.

Title: Supplementary Data 2.
Description: Enriched targets in selected GO Term groups. Lists of associated target genes from the GO Term enrichment analysis are shown for each strain and growth condition for selected significantly enriched GO Terms.

Title: Supplementary Data 3.
Description: Conservation of sRNAs between three *P. aeruginosa* strains. The 200 sRNAs previously identified in PA14 were searched against the PAO1 and IHMA87 genomes. sRNAs with no match are denoted as 'NA' in the corresponding genome.

Title: Supplementary Data 4.
Description: RIP-seq peak calling analysis results. Fold changes and statistical significance values from the PEAKachu analysis for all identified peaks are reported for each strain and growth condition.

Title: Supplementary Data 5.
Description: Intergenic peak calling results. Fold changes and statistical significance values from the PEAKachu analysis for all strictly intergenic peaks are reported for each strain and growth condition.

Title: Supplementary Data 6.
Description: Newly identified putative sRNAs. Lists of all putative sRNAs matching the StructRNAfinder database identified across intergenic peaks for each of the three genomes.

Title: Supplementary Data 7.
Description: Antisense intragenic peak calling results. Fold changes and statistical significance values from the PEAKachu analysis for all antisense intragenic peaks are reported for each strain and growth condition.

Title: Supplementary Data 8.
Description: IHMA87 CRISPR RNA target prediction. Results of the CRISPRCasFinder prediction results for all spacers of the CRISPR gRNA cluster enriched in the IHMA87 Hfq RIP-seq experiment.

Title: Supplementary Data 9.
Description: rGRIL-seq sequencing results. Number of total and vfr-chimeric reads are reported for each strain, condition and replicate.

Title: Supplementary Data 10.
Description: *vfr* chimeraes identified by rGRIL-seq. All genomic features identified in *vfr* chimeric reads and their number are reported for each strain and growth condition.

Title: Supplementary Data 11.
Description: Plasmids and strains used in this study.

Title: Supplementary Data 12.
Description: Primers used in this study.
